# Identification of menstrual psychosis cases using electronic health records

**DOI:** 10.1192/bjp.2025.95

**Published:** 2025-06-01

**Authors:** Thomas J Reilly, Edward Chesney, Adam Al-Diwani, Amelia Jewell, Alexis E Cullen, Dominic Oliver, Philip McGuire

**Affiliations:** 1 Department of Psychiatry, University of Oxford; 2 Department of Psychosis Studies, Institute of Psychiatry, Psychology and Neuroscience, King’s College London; 3 South London and Maudsley NHS Foundation Trust; 4 Department of Addictions, Institute of Psychiatry, Psychology and Neuroscience, King’s College London; 5 Department of Clinical Neuroscience, Karolinska Institutet; 6 NIHR Oxford Health Biomedical Research Centre

Menstrual psychosis is a rare condition characterised by acute episodes of psychosis occurring in synchrony with the menstrual cycle, with complete recovery between episodes.^1^ To date, it has been described in case reports only. We systematically identified cases of menstrual psychosis by examining electronic health records (EHRs) from a large secondary care mental health trust and compared the clinical features with those of a matched control group.

We used the Clinical Record Interactive Search (CRIS) system to search the South London and Maudsley NHS Foundation Trust EHRs.^2^ The project was conducted under CRIS Ethical Approval (Oxfordshire Research Ethics Committee, reference 23/SC/0257). Keywords were used to search the free-text fields of clinical records, for example (premenstrual, menstrual, catamenial, menses) combined with (psychosis, psychotic, mania, manic, bipolar, schizophrenia, schizoaffective). Free-text fields of individuals with a ICD-10 diagnostic codes for psychotic disorders were also searched.

Cases were included if they met Brockington^1^ criteria for menstrual psychosis: (i) acute psychotic episodes; (ii) of less than two weeks in duration; (iii) in synchrony with the cycle; (iv) with full recovery between episodes. Patients with menstrual exacerbations of a chronic psychotic disorder (worsening of psychosis associated with the cycle but not discrete psychotic episodes) were excluded.

Demographic data and clinical outcomes were automatically extracted from structured fields in the health record. Individual symptoms and symptom domains were extracted through manual inspection of the clinical record, using the Association for Methodology and Documentation in Psychiatry (AMDP) symptom checklist.^3^ Up to four controls were identified for each case, from the same set of EHRs. All controls were female; they were then matched by ethnicity, age of illness onset (within a two-year period), and primary ICD-10 diagnosis.

The binary presence/absence of individual symptoms was compared between groups using logistic regression. To reduce a large number of symptoms which were absent in both cases and controls, any symptom with near zero variance was excluded from the analysis.^4^ Symptom domains were pre-specified, taken from the AMDP system. To control for multiple comparisons, the false discovery rate was set at 5% using the Benjamini-Hochberg procedure.^5^

The clinical record search returned 544 unique patient records between 2006 and 2024. Of these, 421 were excluded: 322 had no evidence of psychotic symptoms, 70 had no link with the menstrual cycle and 29 were irrelevant records. Of the 123 records in which a patient, family member or clinician reported a link between symptoms and the cycle, 77 were an exacerbation of an underlying mental disorder, 4 were likely premenstrual dysphoric disorder or premenstrual syndrome, 4 provided insufficient information, and 38 met criteria for menstrual psychosis (diagnosed across 20 different clinical services), forming the case-group for further analyses. There were no statistically significant differences in matching variables, or in IMD score (see Supplementary Table 1). The control group consisted of 112 patients.

Cases had a modal age of onset of 14 years and a median of 17 years (IQR 14-26). The median number of psychotic episodes associated with the cycle was 3 (IQR 2-4). The most common ethnicity was Black (n=15, 39.5%), with most cases being non-White (n=23, 60.5%). The total number of individual symptoms was similar between groups (cases mean 14.6, SD 7.2, controls mean 14.8, SD 6.6, p=0.840). Removing symptoms with near zero variance reduced the total number of symptoms from 153 to 56.

In terms of symptom domains (defined by the presence of at least one individual symptom in that domain), cases had a higher proportion of disorders of consciousness (OR 4.7, 95% 2.1-10.73), but a lower proportion of worries/compulsions (OR 0.34, 95% CI: 0.16-0.73) relative to controls, as shown in Figure 1. In cases, the most common symptoms were auditory verbal hallucinations (n=26, 68.4%), affective lability (n=24, 63.2%), anxiety (n=24, 63.4%), euphoria (n=19, 50%) and motor restlessness (n=19, 50%). After correcting for multiple comparisons, cases were more likely than controls to present with incoherence/derailment (OR 5.48, 95% CI:1.83-17.57), clouded consciousness (OR 4.23, 95% CI: 1.88-9.65) and labile affect (OR 3.09, 95% CI: 1.45-6.76). Conversely, they were less likely to present with suicidal behaviour (OR 0.2, 95% CI: 0.08-0.46), depressed mood (OR 0.21, 95% CI: 0.09-0.45), self-harm (OR 0.24, 95% CI: 0.08-0.62) and suspiciousness (OR 0.32, 95% CI: 0.15-0.69). The full results for both individual symptoms and symptom domains is provided in the Supplementary Material.

Follow-up information was available for 33 (86.8%) cases and 107 (95.5%) controls. There were no group differences in the total number of inpatient admissions, number of inpatient bed-days, or number of clinical contacts (Supplementary table 1).

This EHR study identified 38 possible cases of menstrual psychosis, a condition which had previously only been described in single case reports or small case series. Although auditory verbal hallucinations was the most frequently recorded symptom in cases, the features that most distinguished them from matched controls were incoherence/derailment, clouded consciousness and affective lability. Despite these differences in clinical presentation, cases did not differ from matched controls in clinical outcomes.

The timing of episodes in relation to the menstrual cycle was determined by information documented by clinicians in the health record, and is therefore dependent on the clinician being aware that symptoms may be related to the cycle. Thus, it is likely that the number of cases identified is underestimated and does not reflect the true prevalence of menstrual psychosis. The median number of episodes was small; Brockington^1^ classified cases with 3-4 episodes linked to the cycle as ‘possible’ rather than ‘confirmed’ cases. While we drew controls from the same population and excluded controls with evidence of menstrual exacerbation in their record, we cannot rule out the possibility that some controls had menstrual worsening that was not detected or recorded.

In conclusion, menstrual psychosis is a rare condition which typically emerges in early adolescence, has an episodic course, and is associated with an affective and confusional presentation. Clinicians assessing female patients with psychosis should consider whether the menstrual cycle could be a contributing factor, particularly when it has an early age of onset. Further prospective research into the effect of the menstrual cycle in psychosis is warranted.

## Supplementary Material

Supplementary Materials

## Figures and Tables

**Figure 1:**
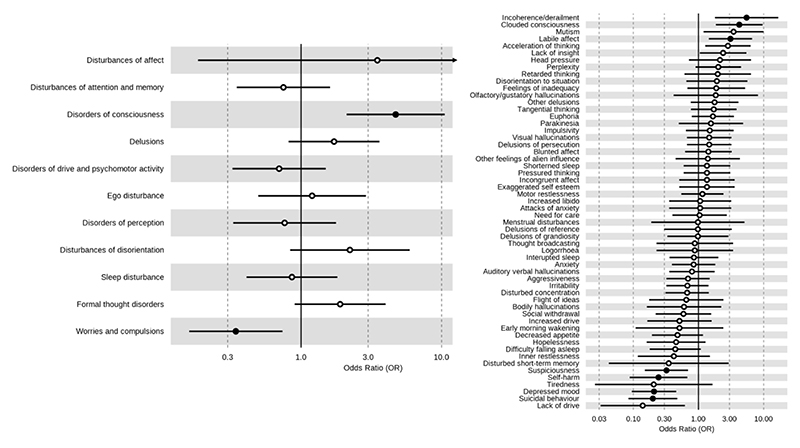
Forest plot of the association of symptom domains (left) and individual symptoms (right) with case-control status. Odds ratio >1 indicates higher proportion in cases, odds ratio <1 indicates higher proportion in controls. Black circle indicates statistical significance corrected at the False Discovery Rate of 5%

## Data Availability

Data are owned by a third party, Maudsley Biomedical Research Centre (BRC) Clinical Records Interactive Search (CRIS) tool, which provides access to anonymised data derived from SLaM electronic medical records. These data can only be accessed by permitted individuals from within a secure firewall (i.e. the data cannot be sent elsewhere), in the same manner as the authors. For more information please contact: cris.administrator@slam.nhs.uk.
