# Supplementary material for: Identification of menstrual psychosis cases using electronic health records
Source: Br J Psychiatry. Author manuscript; Available in PMC 2025 Jul 4. (PMC7617820; doi:10.1192/bjp.2025.95)
Supplement: Supplementary Materials [file EMS204026-supplement-Supplementary_Materials.pdf]

Supplementary material
Identification of menstrual psychosis cases using electronic health records

Thomas J Reilly, Edward Chesney, Adam Al-Diwani, Amelia Jewell, Alexis E Cullen, Dominic Oliver, Philip McGuire

Table of contents

Supplementary tables

Supplementary Tables

Supplementary table 1. Comparison of cases and controls with follow-up data. SD - standard deviations, IMD - index of multiple deprivation, IQR – interquartile range. Groups were compared using t-test for age and IMD score, Chi squared test for Ethnicity and Diagnosis, and Mann-Whitney U test for Admissions, Contacts and Bed days.

	Case (n=33)	Control (n=107)	P value
**Age, mean (SD)**	22.7 (10.8)	24.5 (10.7)	p=0.165
**Ethnicity, number**			
Asian	2 (6.1%)	4 (3.8%)	p=0.943
Black	14 (42.2%)	41 (38.1%)	
Missing	2 (6.1%)	9 (8.4%)	
Mixed race	2 (6.1%)	4 (3.7%)	
Other	1 (3.0%)	5 (4.7%)	
White	12 (36.4%)	44 (41.1%)	
**Diagnosis, number**			
Acute and transient psychotic disorders	9 (27.3%)	30 (28.0%)	p=0.957
Bipolar disorder	12 (36.4%)	32 (29.9%)	
Depressive disorder	2 (6.1%)	7 (6.5%)	
Nonorganic psychosis	6 (18.2%)	25 (23.4%)	
Schizophrenia	4 (12.1%)	13 (12.1%)	
**IMD score, mean (SD)**	23.2 (8.9)	26.0 (10.4)	p=0.168
**Clinical outcomes**			
Admissions, median (IQR)	2 (2)	2 (4)	p=0.660
Contacts, median (IQR)	54 (124)	46 (122)	p=0.547
Bed days, median (IQR)	131 (118)	101 (242)	p=0.971

*Supplementary table 2. Logistic regression of symptom domains by case-control status. P values are corrected for multiple comparisons using the Benjamini-Hochberg procedure. OR – odds ratio, 95% CI – 95% confidence interval. *indicates statistical significance at false discovery rate of 5%. Proportion of cases with Disturbances of affect reduced to 0.99 to allow calculation of OR.*

	Proportion				
**Symptom domain**	**Cases (n=38)**	**Controls (n=112)**	**OR**	**95% CI**	**Corrected p value**
Disturbances of affect	0.99	0.96	3.5	0.37 – 466.5	0.628
Disorders of perception	0.74	0.79	0.76	0.33 - 1.85	0.736
Disorders of drive and psychomotor activity	0.61	0.69	0.7	0.33 - 1.51	0.649
Formal thought disorders	0.58	0.42	1.9	0.91 - 4.06	0.305
Sleep disturbance	0.58	0.62	0.86	0.41 - 1.83	0.755
Delusions	0.53	0.39	1.72	0.82 - 3.63	0.337
Disorders of consciousness	0.47	0.16	4.7	2.1 - 10.73	<0.001*
Worries and compulsions	0.47	0.72	0.34	0.16 - 0.73	0.033*
Disturbances of attention and memory	0.37	0.44	0.75	0.35 - 1.58	0.718
Ego disturbances	0.24	0.21	1.2	0.48 - 2.82	0.755
Disturbances of disorientation	0.21	0.11	2.22	0.8 - 5.89	0.305

*Supplementary table 3. Logistic regression of individual symptoms by case-control status. P values are corrected for multiple comparisons using the Benjamini-Hochberg procedure. OR – odds ratio, 95% CI – 95% confidence interval. *indicates statistical significance at false discovery rate of 5%*

Symptom	Proportion Cases (n=38)	Proportion Controls (n=112)	OR	95% CI	Corrected p value
Clouded consciousness	0.45	0.16	4.23	1.88 - 9.65	0.019*
Disorientation to situation	0.16	0.09	1.91	0.61 - 5.57	0.576
Disturbed concentration	0.34	0.44	0.67	0.3 - 1.42	0.594
Disturbed short-term memory	0.03	0.07	0.35	0.02 - 2.01	0.594
Retarded thinking	0.13	0.07	1.97	0.56 - 6.33	0.587
Pressured thinking	0.29	0.23	1.35	0.57 - 3.04	0.672
Flight of ideas	0.08	0.12	0.65	0.14 - 2.17	0.706
Tangential thinking	0.34	0.23	1.72	0.76 - 3.81	0.501
Incoherence/derailment	0.24	0.05	5.48	1.83 - 17.57	0.037*
Suspiciousness	0.45	0.71	0.32	0.15 - 0.69	0.037*
Delusions of reference	0.11	0.11	0.98	0.26 - 3.03	0.982
Delusions of persecution	0.37	0.29	1.46	0.66-3.15	0.594
Delusions of grandiosity	0.13	0.13	0.98	0.3 - 2.75	0.982
Other delusions	0.29	0.19	1.77	0.74 - 4.07	0.501
Auditory verbal hallucinations	0.68	0.73	0.79	0.36 - 1.81	0.709
Visual hallucinations	0.37	0.29	1.46	0.66 - 3.15	0.594
Bodily hallucinations	0.08	0.12	0.6	0.13 - 1.97	0.67
Olfactory and gustatory hallucinations	0.08	0.04	1.83	0.36 - 7.87	0.656
Thought broadcasting	0.08	0.09	0.87	0.19 - 3.05	0.946
Other feelings of alien influence	0.13	0.1	1.39	0.41 - 4.13	0.709
Perplexity	0.34	0.21	2.01	0.88 - 4.51	0.392
Blunted affect	0.29	0.22	1.42	0.6 - 3.21	0.656
Depressed mood	0.39	0.76	0.21	0.09 - 0.45	<0.001*
Hopelessness	0.13	0.25	0.45	0.14 - 1.19	0.473
Anxiety	0.63	0.67	0.85	0.4 - 1.85	0.814
Euphoria	0.5	0.38	1.67	0.79 - 3.52	0.501
Irritability	0.47	0.57	0.67	0.32 - 1.41	0.594

Inner restlessness	0.08	0.17	0.42	0.09 - 1.33	0.501
Feelings of inadequacy	0.18	0.11	1.88	0.65 - 5.11	0.565
Exaggerated self-esteem	0.18	0.14	1.35	0.48 - 3.49	0.706
Incongruent affect	0.18	0.14	1.35	0.48 - 3.49	0.706
Affective lability	0.63	0.36	3.09	1.45 - 6.76	0.037
Lack of drive	0.05	0.29	0.14	0.02 - 0.49	0.063
Increased drive	0.11	0.19	0.51	0.14 - 1.46	0.576
Motor restlessness	0.5	0.46	1.15	0.55 - 2.42	0.825
Parakinesia	0.13	0.09	1.55	0.45 - 4.69	0.671
Mutism	0.21	0.07	3.47	1.18 - 10.2	0.123
Logorrhoea	0.08	0.09	0.87	0.19 - 3.05	0.946
Social withdrawal	0.16	0.24	0.59	0.21 - 1.48	0.594
Aggressiveness	0.34	0.43	0.69	0.31 - 1.47	0.594
Suicidal behaviour	0.21	0.57	0.2	0.08 - 0.46	<0.001*
Self-harm	0.13	0.38	0.24	0.08 - 0.62	0.048
Lack of insight	0.34	0.18	2.39	1.03 - 5.46	0.199
Need for care	0.18	0.18	1.04	0.38 - 2.6	0.982
Attacks of anxiety	0.13	0.12	1.06	0.32 - 3.01	0.982
Acceleration of thinking	0.39	0.19	2.83	1.26 - 6.34	0.068
Impulsivity	0.29	0.21	1.49	0.63 - 3.39	0.594
Difficulty falling asleep	0.18	0.34	0.44	0.17 - 1.04	0.355
Interrupted sleep	0.24	0.27	0.85	0.34 - 1.95	0.825
Shortened sleep	0.29	0.23	1.35	0.57 - 3.04	0.672
Early morning wakening	0.05	0.1	0.51	0.08 - 2.02	0.652
Tiredness	0.03	0.12	0.21	0.01 - 1.09	0.473
Decreased appetite	0.18	0.32	0.48	0.18 - 1.13	0.444
Menstrual disturbances	0.05	0.05	0.98	0.14 - 4.48	0.982
Head pressure	0.16	0.08	2.15	0.67 - 6.42	0.501
Increased libido	0.13	0.12	1.06	0.32 - 3.01	0.982
